# The Impact of Subsidies on the Ecological Sustainability and Future Profits from North Sea Fisheries

**DOI:** 10.1371/journal.pone.0020239

**Published:** 2011-05-26

**Authors:** Johanna Jacomina Heymans, Steven Mackinson, Ussif Rashid Sumaila, Andrew Dyck, Alyson Little

**Affiliations:** 1 Scottish Association for Marine Science, Scottish Marine Institute, Oban, Argyll, United Kingdom; 2 Cefas, Lowestoft, Suffolk, United Kingdom; 3 UBC Fisheries Centre, Vancouver, British Columbia, Canada; 4 School of Biological Sciences, University of Aberdeen, Aberdeen, United Kingdom; Institute of Marine Research, Norway

## Abstract

**Background:**

This study examines the impact of subsidies on the profitability and ecological stability of the North Sea fisheries over the past 20 years. It shows the negative impact that subsidies can have on both the biomass of important fish species and the possible profit from fisheries. The study includes subsidies in an ecosystem model of the North Sea and examines the possible effects of eliminating fishery subsidies.

**Methodology/Principal Findings:**

Hindcast analysis between 1991 and 2003 indicates that subsidies reduced the profitability of the fishery even though gross revenue might have been high for specific fisheries sectors. Simulations seeking to maximise the total revenue between 2004 and 2010 suggest that this can be achieved by increasing the effort of Nephrops trawlers, beam trawlers, and the pelagic trawl-and-seine fleet, while reducing the effort of demersal trawlers. Simulations show that ecological stability can be realised by reducing the effort of the beam trawlers, Nephrops trawlers, pelagic- and demersal trawl-and-seine fleets. This analysis also shows that when subsidies are included, effort will always be higher for all fleets, because it effectively reduces the cost of fishing.

**Conclusions/Significance:**

The study found that while removing subsidies might reduce the total catch and revenue, it increases the overall profitability of the fishery and the total biomass of commercially important species. For example, cod, haddock, herring and plaice biomass increased over the simulation when optimising for profit, and when optimising for ecological stability, the biomass for cod, plaice and sole also increased. When subsidies are eliminated, the study shows that rather than forcing those involved in the fishery into the red, fisheries become more profitable, despite a decrease in total revenue due to a loss of subsidies from the government.

## Introduction

Fisheries subsidies can be categorised as beneficial, capacity-enhancing or ambiguous. Beneficial subsidies are programs that lead to investment in natural capital such as fish stocks. Capacity-enhancing subsidies lead to disinvestments in natural capital assets that lead to overexploitation and remove the ability of the fishery to be sustainable in the long term. Ambiguous subsidies are those whose impact are undetermined and could lead to either investment or disinvestment in the fishery resource [1]. Capacity-enhancing subsidies are the most harmful and include fuel subsidies, boat construction, renewal and modernisation programs, fishing port construction and renovation programs, price and marketing support, processing and storage infrastructure programs, fishery development projects, tax exemptions and foreign access agreements [1]. Most subsidies provided by many governments around the world are harmful, amounting to US$16.2 billion out of a total of US$27 billion a year globally, while beneficial subsidies amount to only US$ 8 billion [1]. Europe is second only to Asia in subsidy provision, at US$ 4.7 billion, which is about 56% of Europe's catch value [1].

Harmful fisheries subsidies negatively affect the long-term sustainability of the ecosystem (because they lead to overcapacity), which is already under threat from climate change [2], invasive species and pollution [3]. Fishing subsidies have come under increasing scrutiny from conservationists and politicians alike. For example, it has been shown to be the only way whaling can still be undertaken in Norway and Japan [4]. In the Black Sea, subsidies such as tax credits, import tax exemptions on equipment and on construction material are described as drivers of higher pressure, and are shown to relate to increases in total engine power [5]. Globally, the fishing industry is being subsidised each year by billions of dollars to continue fishing: governments are therefore effectively funding over-exploitation of marine resources [1], [6]. This over-exploitation has had a detrimental effect on the productivity of fisheries and the reorganization of the ecosystem over the past 100 years [7], [8] and has been funded by subsidies for at least 55 years [9]. However, the EU Common Fisheries Policy aims to ensure exploitation of living aquatic resources that provides sustainable economic, environmental and socially ethical fisheries [10] and as such, the impact of subsidies needs to be explicitly examined.

**Figure 1:**
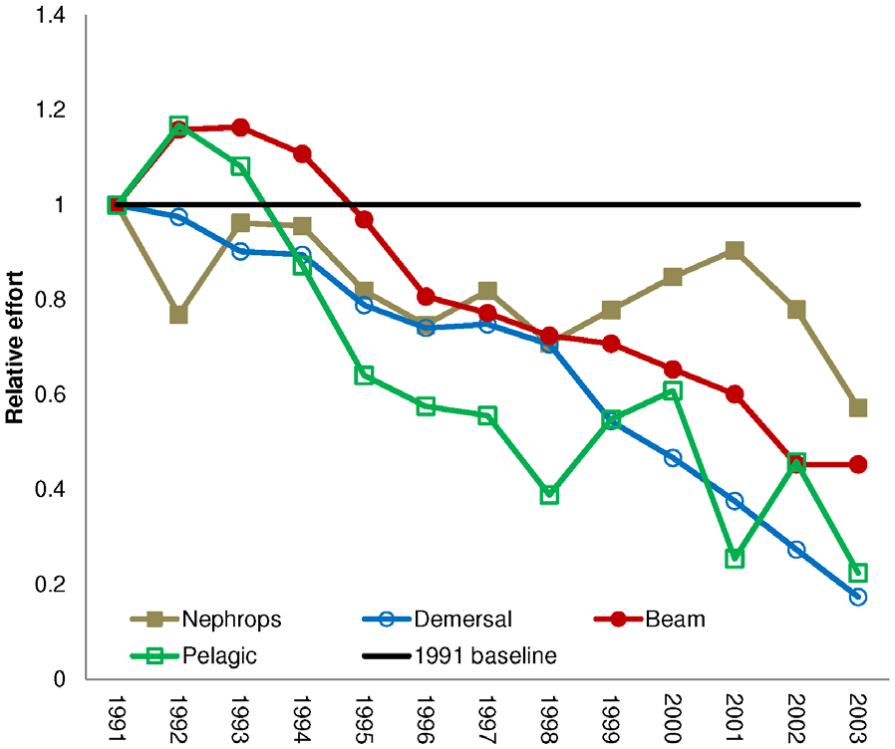
Trends in relative effort of the modelled fishing fleet, standardized to 1 in 1991. Change in effort of the Nephrops trawlers, demersal trawl-and-seine fleets, beam trawlers, and the pelagic trawl-and-seine fleet relative to the effort for each of these fleets observed in 1991 (1991 baseline). Data obtained from ICES WG assessment reports defined in Table S4, where effort is given in hours fished. Effort of most fleets show a reduction over the 14 years modelled, with the pelagic and beam trawlers showing some increase in the first few years followed by a decline until 2003.

The major fishing nations in the North Sea are Denmark, the UK, the Netherlands and Norway, with Germany, Belgium and France also active in the fishery. The principal fishing fleets (Figure 1) are industrial and target several demersal and pelagic species. These fleets are subsidised by their countries to varying degrees. A crucial step to helping the EU and the relevant countries to reduce harmful fisheries subsidies is to demonstrate the impacts these subsidies have on the health of the ecosystem and the economic and social wellbeing of the fishing sector in Europe. To date, most of the discussion on the effects of fisheries subsidies on sustainability is based on theoretical models [1], [11], [12].

The aim of this study is to investigate the impacts of fisheries subsidies on the ecological resilience and economic profitability of the North Sea ecosystem. This will be achieved by using an ecosystem model to contrast how policies on subsidies might influence fleet structure in terms of relative effort of the principal fleets, and therefore the economic and social contribution to the wellbeing of European fisheries. The model will also be used to examine the impact of subsidies on the optimisation for maximum profit vs ecological stability.

## Results

Results from the two analyses are given below: 1) Profits obtained from the hindcast analysis of the published, fitted, ecosystem model [13], [14] from 1991–2003 compared to the model where subsidies were eliminated; and 2) Simulations from 2003–2010 where the model was optimised for maximum profit or maximum ecological stability – including “with subsidies” and “without subsidies” scenarios - to test the impact of subsidies as well as objective functions on the profit, fisheries stability and resilience of the ecosystem.

### 1. Hindcasting

**Figure 2:**
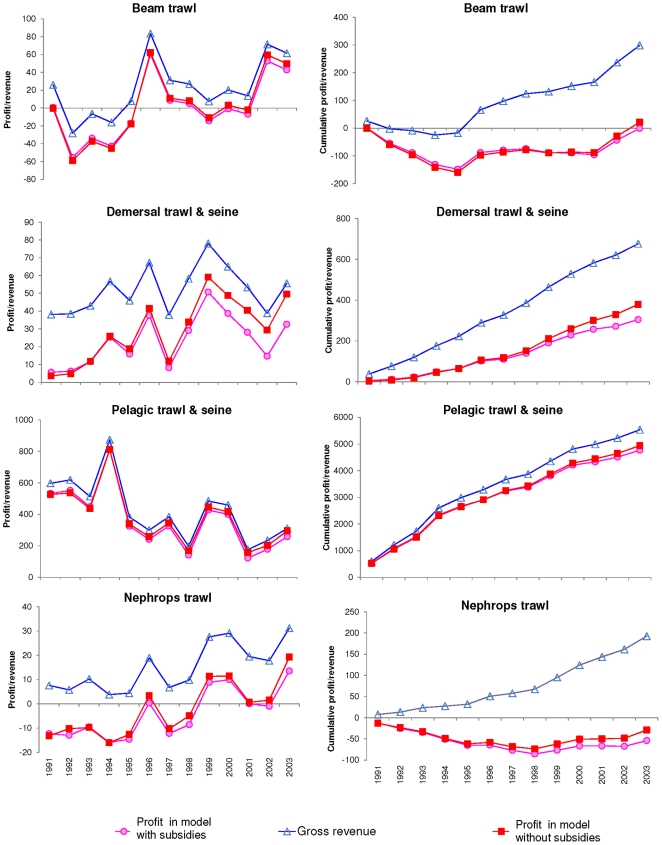
Profits, cumulative profit and revenue obtained with and without subsidies (in € million). Profits (pink) and gross revenue (blue) in the “with subsidies” model, pelagic trawl and seine fleet (2E and 2F) and the Nephrops trawlers (2G and 2H), with subsidies and profit when subsidies were removed from the model (red). All left hand figures show true values and right hand figures show cumulative values - all in € million. In all cases gross revenue is higher than profit because costs are subsidised. Both the demersal (2C, 2D) and pelagic fleets (2E, 2F) were profitable for the whole time series, although the demersal trawlers profitiability showed an upward trend while the pelagic fleet profitability declined. However, the initial difference in profits for demersal and Nephrops fleets seem large but that is due to the scale of their profits compared to that of the pelagic fleet. The differences between gross revenue (square) and profit in the model without subsidies (red) diminish over the 12 years of the simulation due to the fact that the effort for all these fleets decline over time (Figure 1), which reduces the variable (effort related) cost in the Ecopath model without subsidies. The beam trawlers (2A, 2B) became profitable only when effort declined substantially, because of the reduction in effort reduces the variable costs. Similarly, the Nephrops trawlers (2G, 2H) became profitable in 1999, although cumulatively they had still not shown a profit by 2003, even though their gross revenue increased over time.

The variable costs of each fleet change with changes in effort, and as such only those fleets with changes in effort will show changes in variable cost over time. These changes in effort cause changes in the profit made by each fleet, with the pelagic fleet starting off with the biggest profit, and also the largest difference between subsidised and non-subsidised profit (Figure 2). Figure 2 shows the profit and gross revenue (left) as well as the cumulative profit (right) for each fleet over time (in € millions). Figure 2 also shows the profit (when subsidies are removed from the profit calculated by Ecosim, pink) and the gross revenue that the fishers have taken home over time (blue). Finally, in the model where subsidies were removed from the value of the fishery, the estimated profit is also shown (red).

The initial difference for demersal and beam fleets seem large but that is due to the scale of their profits compared to that of the pelagic fleet. In addition, the profit with subsidies (pink) does not seem much lower than that without subsidies (red), but for example in 2003 the profit without subsidies of beam trawlers (Figure 2A) was € 50 million, while that with subsidies was € 43 million - a difference of € 7 million - while the gross revenue was € 62 million – thus the governments of the North Sea paid an extra €19 million to make the beam trawler fisheries less profitable by €7 million in that year and cumulatively the beam trawler fishery was in a deficit of €1 million from 1991–2003, while they could have accumulated a profit of €21 million without subsidies (Figure 2B).

From Figure 2, it seems that the differences between gross revenue (blue) and profit in the model without subsidies (red) diminish over the 12 years of the simulation. This is due to the fact that the effort for all these fleets decline over time (Figure 1), which reduces the variable (effort related) cost in the Ecopath model without subsidies. The beam trawlers became profitable (Figure 2A) only when effort declined substantially, i.e. 1996 and 2002 (Figure 1) because the reduction in effort reduces the variable costs in those two years.

The beam trawlers start off at a loss in 1991 and cumulatively make a loss for the whole simulation (red), except for the last year, although their gross revenue was above zero from 1995 onwards (blue). Similarly, the cumulative profits of Nephrops trawlers (Figure 2H) are also never positive (i.e. both these fleets are working at a loss) over the 12 years from 1991 to 2003, but the gross revenue was positive for all of the simulation. Without subsidies, the Nephrops fleet makes losses year on year until 1998, when the effort decreased substantially (Figure 2G). After 1998 the effort increases again and the cumulative profit starts to increase, although the fleet was still losing money by the end of the simulation (2003).

In all cases gross revenue is higher than profit because costs are subsidised. However, the profit of the demersal and pelagic trawls and seines are minimised with the reduction in effort, while that of the beam trawl increases over the time period of the simulation and the Nephrops trawl profit declines.

### 2. Optimisation

In this analysis the model with and without subsidies are simulated forward by optimising for maximum profit or maximum ecological stability. Here, we define ecological stability as the longevity-weighted summed biomass for all the ecosystem groups, following Odum's [15], [16] definition of ecosystem maturity [17] and by definition stability, by assuming that ecosystems with many long lived animals will be more stable.

**Figure 3:**
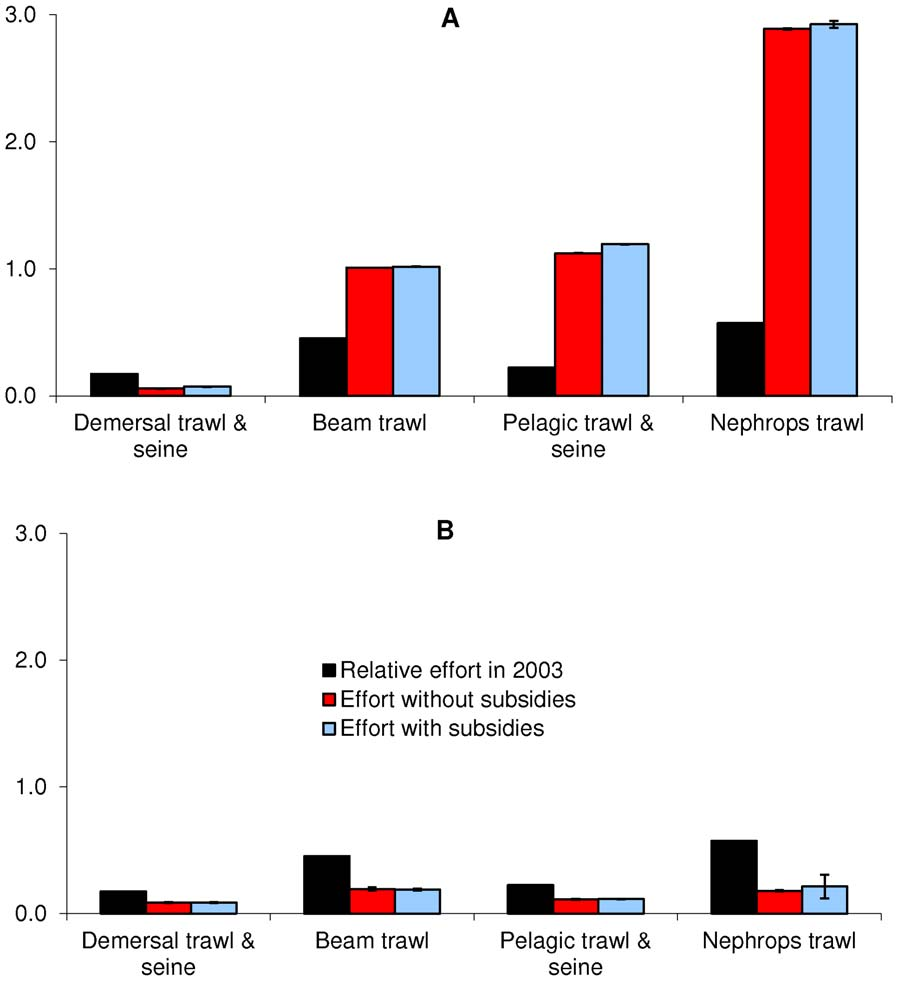
Relative effort (+ standard deviation), estimated, when optimising for A) profit and B) ecological stability. Effort in 2003 relative to the 1991 basline and those estimated by the policy optimisation routine in models with and without subsidies when optimising for A) profit and B) ecological stability. Figure 3A shows that, when optimising for profits, the effort of the demersal fleets declined significantly regardless of whether subsidies were applied or not, while beam, pelagic and Nephrops fleets increased. This is because the profit that can be made given the prices of the species caught by these fleets is much lower for the demersal fleets than for the Nephrops fleets. Nephrops command a high ex-vessel price (Table S5), so it is unsurprising that the optimisation seeks to maximise effort and yield from this fleet. The effort of all fleets was slightly higher when subsidies were included (Figure 3A). This is because the cost of fishing is lower when subsidies are included, and so more effort can be expended for the same cost. When optimising for ecological stability (Figure 3B) the relative effort will have to decrease significantly from that of 2003, and that effort with subsidies will be marginally higher than without.

The profit optimisation runs showed that after 2003 the effort of the demersal fleets declined significantly regardless of whether subsidies were applied or not, while beam, pelagic and Nephrops fleets increased (Figure 3A). The difference between effort with and without subsidies might seem insignificant when compared to changes in effort by fleet when optimising for profit (Figure 3A), but in the 10 simulations the minimum effort with subsidies always exceeded the maximum effort without subsidies. The differences in effort by fleet is because the profit that can be made given the prices of the species caught by these fleets is much lower for the demersal fleets than for the Nephrops fleets. Nephrops command a high ex-vessel price (Table S5), so it is unsurprising that the optimisation seeks to maximise effort and yield from this fleet. The effort of all fleets was slightly higher when subsidies were included (Figure 3A). This is because the cost of fishing is lower when subsidies are included, and so more effort can be expended for the same cost. Figure 3B shows that when optimising for ecological stability the relative effort will have to decrease significantly from that of 2003, and that effort with subsidies will be marginally higher than without.

**Figure 4:**
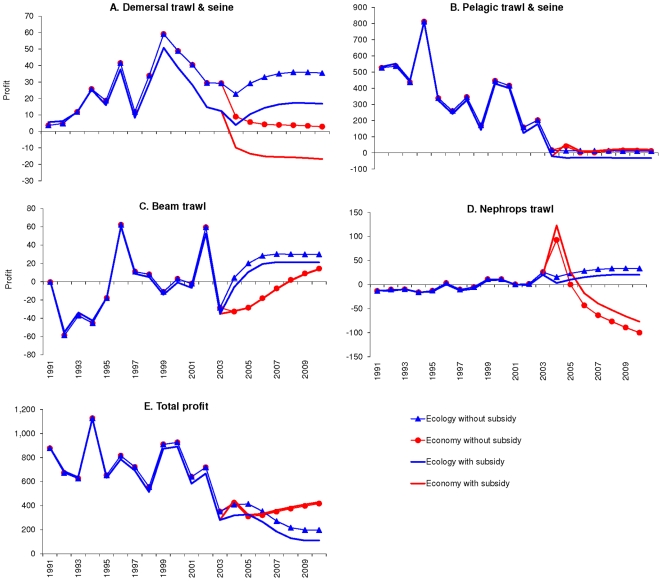
Profits (in € million) with and without subsidies when optimising for profit or ecological stability. When optimising for profit (economy, red) or ecological stability (blue) from 2003 forward to 2010, with or without subsidies, the profits (in € million) were substantially different. Optimising for profit showed that the Nephrops fleet (Figure 4D) became the most profitable fleet in the system in 2004 due to the large increase in its effort (Figure 3A). Despite the increased effort, profits were not sustained over the period simulated, and the fleet goes into a loss in the last 5 years even with subsidies. The profit obtained when subsidies are included are dramatically less for the demersal trawlers than when no subsidies are given (Figure 4A), while the profit for the Nephrops trawlers seems to increase when subsidies are included. By contrast, when optimising for ecological stability (blue lines in Figure 4), all fisheries would do better if no subsidies are given. When optimising for ecological stability, the profit for the demersal, beam and Nephrops trawls increase marginally and stabilise over time at values similar to that of the early 2000s (Figure 4D). These profits are obtained by reducing the effort of most fleets (Figure 3B), and therefore the landings of most species specifically in the first year of the simulation (2004). The total profit obtained from the fisheries (Figure 4E) when optimising for profit overtakes that obtained from optimising for ecological stability in 2006 and when optimising for profit. When optimising for ecological stability, subsidising the fishery will decrease the profitability of the fishery.

**Figure 5:**
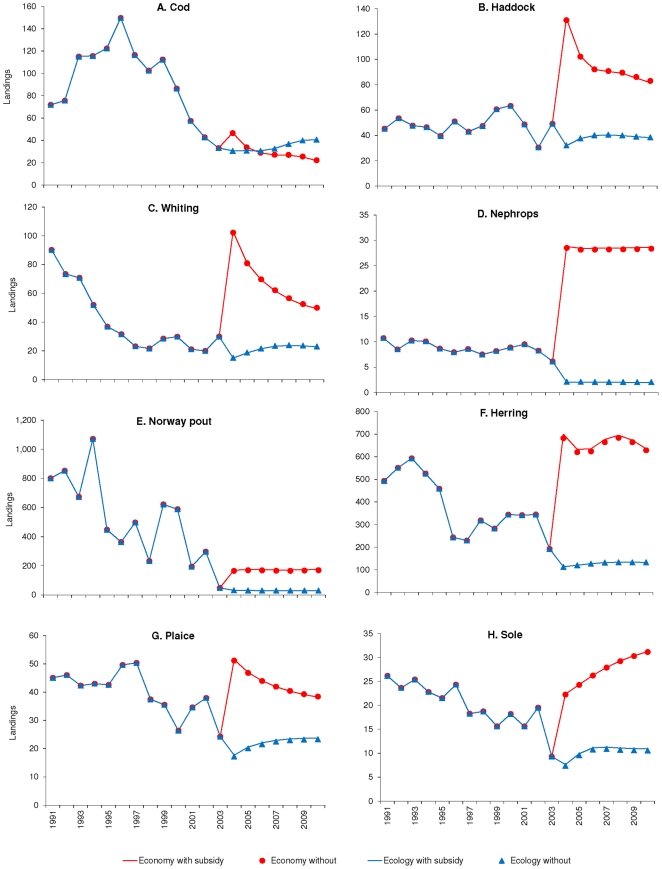
Annual landings (in 1000 tonnes) with and without subsidies when optimising for profit and ecological stability. Annual landings (1000 tonnes) of A. cod, B. haddock, C. whiting, D. Nephrops, E. Norway pout, F. herring, G. plaice and H. sole estimated when optimising for profit (Economy, red) and ecological stability (blue), with and without subsidies. The increase in effort by the Nephrops fleet when optimising for profit (Figure 3A) increase the landings of that species, but also has an impact on the landings of cod, haddock, whiting, herring and plaice all of which are bycatch species in the Nephrops fishery, and those speies are not able to withstand the higher effort as Nephrops could. When optimising for ecological stability, the reduced effort in all fleets (Figure 3B) cause the landings of most species to increase over time, as they recover from the prior higher fishing pressure. However, the landings of lower trophic level species such as Nephrops, Norway pout and herring do not recover as quickly, probably due to the higher predation pressure on those species.

**Figure 6:**
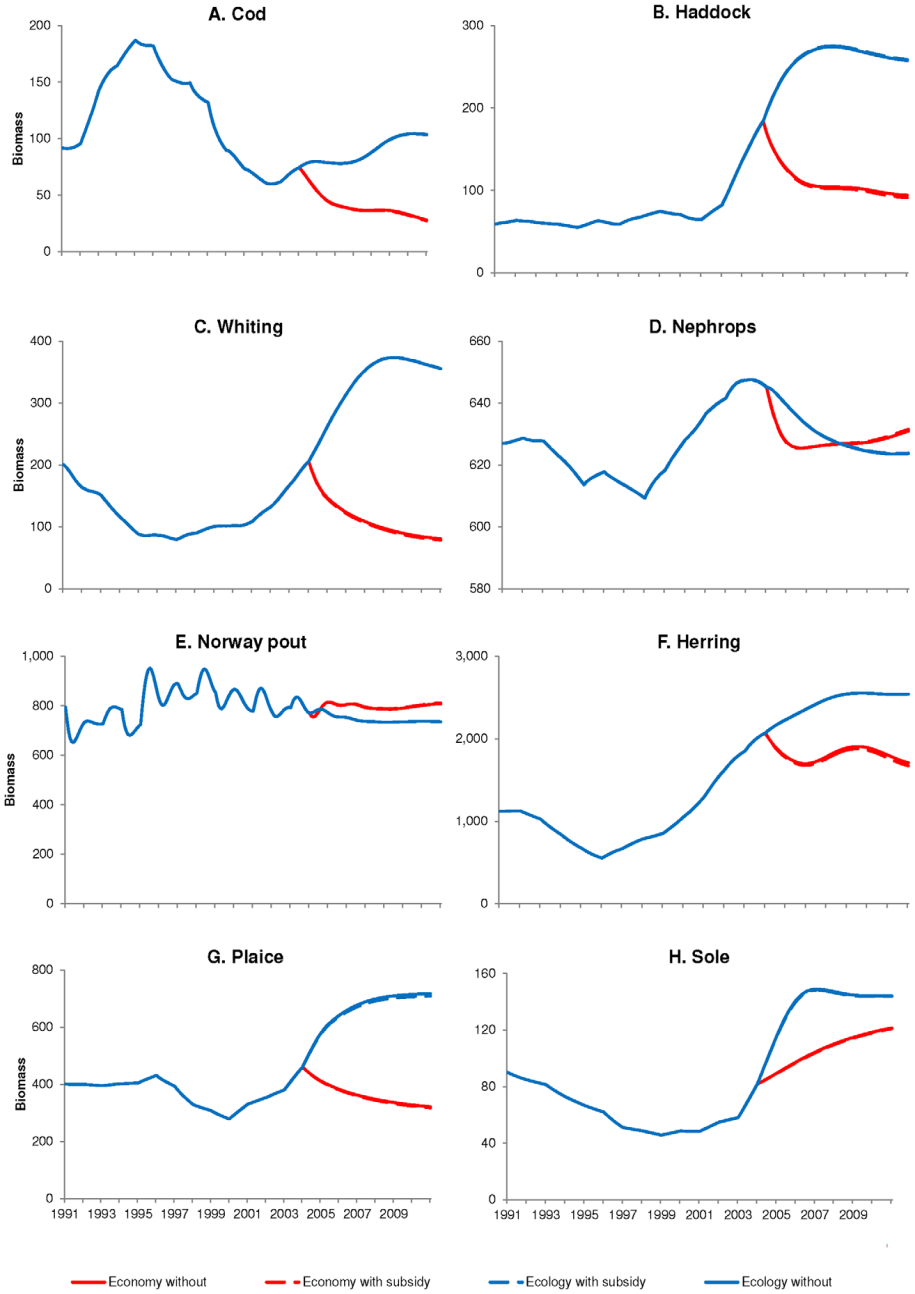
Changes in biomass (in 1000 tonnes) when optimising for profit or ecological stability, with and without subsidies. The biomass (1000 tonnes) of A. cod, B. haddock, C. whiting, D. Nephrops, E. Norway pout, F. herring, G. plaice and H. sole. The biomass of hake, haddock, whiting, Nephrops, Norway pout, herring, plaice and sole showed very little difference when optimising with or without subsidies. The main changes occurred when optimising for profit, where the increase in Nephrops trawl effort (Figure 3A) cause a large decline in the biomass of its target species (Nephrops) as well as all its bycatch species (cod, haddock, whiting, herring and plaice). The initial decline in Nephrops was stabilised while Norway pout biomass increased during the simulation. The reduction in effort when optimising for ecological stability caused the biomass of most species to increase over time, except for Nephrops and Norway pout, again two species that are prey for many of the larger predatory species that were protected by the reduction in effort.

The Nephrops fleet is the most profitable fleet in the system. Despite the increased effort (increased 3 times, Figure 3), profits are not sustained over the period simulated, and the fleet goes into a loss in the last 4 years even with subsidies (Figure 4D). This is because profits to the Nephrops fleet does not only come from Nephrops catches, but also from other species caught and sold by that fleet (see catch composition in Table S3). The declines observed are due to loss of catch for whiting, haddock and plaice, all of which are also caught by the Nephrops trawl. This demonstrates the tradeoffs among fleets as all three species are targeted by other fleets (demersal and beam trawlers). The increase in Nephrops fleet effort increases the fishing mortality on Nephrops and therefore their landings (Figure 5D). However, it also increases the fishing mortality on other species that are caught by the Nephrops trawl, such as whiting, haddock and plaice (Table S5). Specifically the landings of plaice (Figure 5G) whiting (Figure 5C) and haddock (Figure 5B) increase significantly in the first year of the policy optimisation, but both species are not able to sustain the higher fishing mortality from the Nephrops trawl. Therefore the biomass of both species declines (Figures 6B, C, G), causing their total landings to decline and thus the total value of the Nephrops trawl declines. By contrast, the landings of herring (Figure 5F) and sole (Figure 5H) both increase (for herring rather dramatically) but their biomass are not substantially depleted, while the biomass of sole increases over the simulation period. The herring biomass will also be dependent on changes in primary production as they feed lower down the food web, and as all the environmental drivers are kept constant this result has to be taken with that caveat in mind.

The profit obtained when subsidies are included are dramatically less for the demersal trawlers than when no subsidies are given (Figure 4A), while the profit for the Nephrops trawlers seems to increase when subsidies are included. By contrast, when optimising for ecological stability (blue lines in Figure 4), all fisheries would do better if no subsidies are given. When optimising for ecological stability, the profit for the demersal, beam and Nephrops trawls increase marginally and stabilise over time at values similar to that of the early 2000s (Figure 4A). These profits are obtained by reducing the effort of most fleets (Figure 3), and therefore the landings of most species specifically in the first year of the simulation (2004). Some of the landings increase over time, specifically for cod, whiting, plaice and sole (Figure 5) as their biomasses recover (Figure 6).

Conversely the landings of Nephrops, herring and Norway pout stays low (Figure 5), and only the biomass of herring seems to be recovering in this simulation (Figure 6). Norway pout and Nephrops are important in the diet of many species, thus any optimisation that increases the biomass of their predators would be detrimental to the biomass of these two species.

When optimising for ecological stability the profitability of some fleets are maximised because optimising for ecological stability reduces the landings of species caught by the demersal fleets, beam trawlers and Nephrops trawlers, which causes and increase in their biomass. Many of these species are very profitable, such as sole, turbot, lemon sole, monkfish, hake and halibut. These gears discard some of these profitable species and the juveniles of some of the main commercial species such as cod, haddock and whiting, which reduces the ability for the juveniles to grow into adults and be caught in later years. Thus reducing the effort will increase the biomass of these species over time (as seen in Figure 6) and therefore increase the profitability of these gears. This is one of the perverse feedbacks in ecosystems that need to be taken into consideration when managing ecosystems.

### 3. Ecosystem impacts

**Figure 7:**
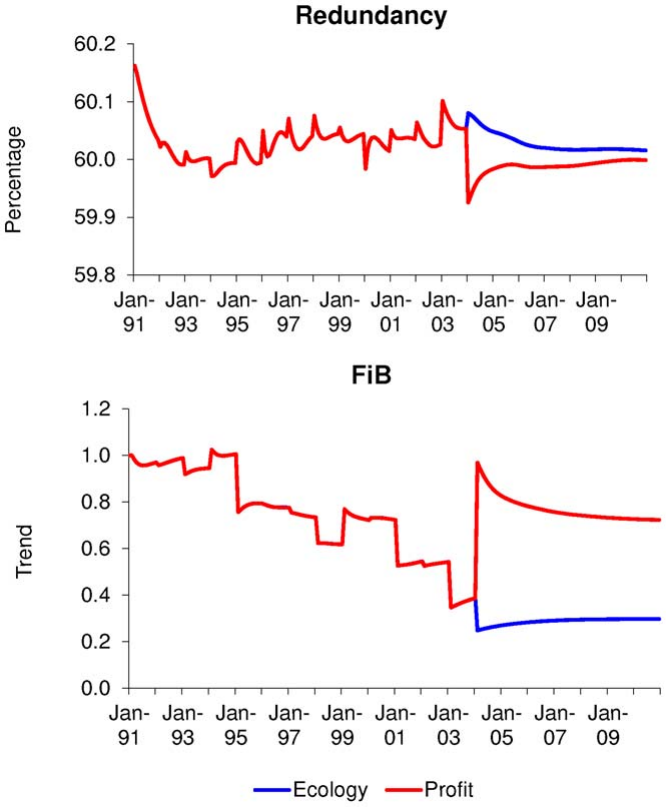
Ecosystem redundancy and “Fisheries in Balance” indices estimates when optimising for profit or ecological stability. The stability of the fishery (described by the Fisheries in Balance index, or FiB) and the ecosystem redundancy are described in Figure 7. The ecosystem indices do not show any significant differences between the scenario with and without subsidies, but do show the impact of the large change in the different fleets in year 14. The different impacts of optimising for profit vs. ecological stability are also shown (Figure 7), with the redundancy of the system being negative affected by optimising for profit, while it is improved by optimising for the ecological stability. The large increase in the Nephrops trawl effort significantly reduces the redundancy and the structure of the ecosystem in year 14 and the ecosystem does not regain its resilience in the remaining 6 years of the simulation. The FiB show a large jump with the much larger catch of Nephrops, which is quite a low trophic level species, but it is reduced when optimising for ecological stability.

The fishery stability (described by the fisheries in balance index, or FiB) and ecosystem redundancy are described in Figure 7. The ecosystem indices do not seem to show any significant differences between the scenario with and without subsidies, but do show the impact of the large change in the different fleets in 2004 – the first year of the optimisation. The different impacts of optimising for profit vs. ecological stability are also shown (Figure 7), with the redundancy of the system being negatively affected by optimising for profit, while it is improved by optimising for the ecological stability. The large increase in the Nephrops trawl effort significantly reduces the redundancy and the structure of the ecosystem in 2004 and the ecosystem does not regain its resilience in the remaining 6 years of the simulation. The FiB show a large jump with the much larger catch of Nephrops, which is quite a low trophic level species, but it is reduced when optimising for ecological stability.

Finally, The results show that in the short term (the 7 years of these simulations) the objective of management matters more than whether subsidies are provided or not. Thus, if the objective is to optimise ecosystem longevity as oppose to maximizing for profit, the fleet structure would be very different. However, if you are optimising for profit, then having capacity enhancing subsidies would increase fishing effort, but not ‘true’ profit.

**Figure 8:**
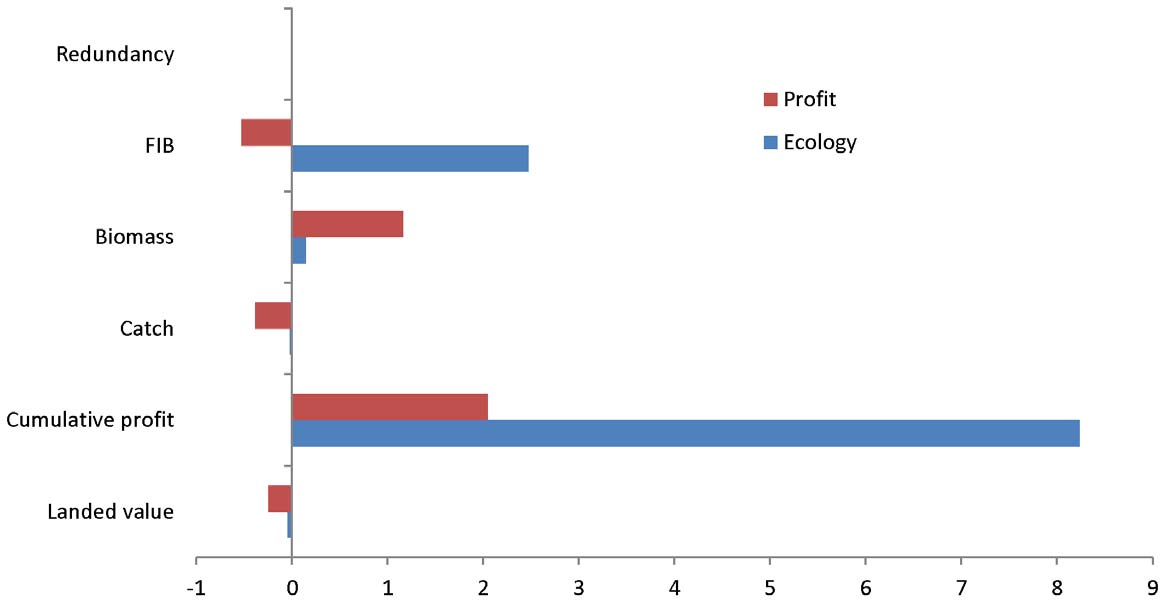
Percentage increase in ecosystem indices when optimising for profit or ecological stability without subidies. The percentage difference in ecosystem redundancy, FiB, and the biomass of cod, haddock, whiting, Nephrops, Norway pout, plaice and sole at end of the simulation (2010) and the total catch, cumulative profit and landed value of all species between 1991–2010 when subsidies were excluded and optimising for profit or ecological stability. Positive values indicate that removing subsidies would increase values, such as that of cumulative profit and biomass. Negative values indicate that excluding subsidies have a negative impact, such as the reduction in landed value obtained without subsidies. The large increase in cumulative profit without subsidies when ecological stability is the objective function shows the importance of removing subsidies to the profitability of the fisheries.

The impact of subsidies on the ecosystem indicators such as redundancy, FiB total biomass of important species, total catch, cumulative catch and landed values is depicted in Figure 8 which shows the percentage difference in these indices without subsidies when optimising for profit or ecological stability. Without subisidies the cumulative profit of the fishery when optimising for ecological stability would be 8% higher, while when optimising for profit it would have been 2% higher. In addition, the fishery would have been more balanced (positive FiB) when optimising for ecological stability, while optimising for profit without subsidies would cause the fishery to change dramatically and give a negative FiB (Figure 8).

It is clear that the subsidies have a larger impact on the fleet's financial performance (profit and landings) than on the ecological indicators such as redundancy. This is because it is easy to increase the biomass of some species in 7 years, and therefore the landings of these species, but not as easy to increase the longevity of all species – which would be needed to improve the ecological longevity of the ecosystem. This shows that if one wants to manage species sustainably one needs to take the long term perspective and it would take more than the 7 years of our simulation to undo 200 years of intensive fishing.

## Discussion

At an EU seminar on financial policy in the future Common Fisheries Policy in Brussels on the 13^th^ of April 2010, Magnus Eckeskog of the Fisheries Secretariat of Sweden concluded that “In order to be able to assess which EU subsidies are good for the environment, we need a full assessment of all EU fisheries subsidies and their impacts on the environment.” This study is a first step towards that end in the North Sea.

Stouten et al. [18] observed high non-linearity of complex systems resulting in unexpected behaviour. They found that fisheries management plans do not always work as expected, and that models can provide managers with a likely range of outcomes to take into account the complexity and feedback within the system [18] in [19]. The general tendency in resource management is to misperceive feedbacks and the workings of stock and flow relationships and insensitivity to the nonlinearities that may alter the strengths of different feedback loops in the system [20]. Moxnes [20] found that misperceptions of feedback can be more devastating to human decision making than biases and that even when fishery managers know that there is uncertainty in the stock and recruitment measurements they would still over-invest in the fishery.

The results from the optimisations show that in spite of higher landed values and catches with subsidies (indicated by negative values for landed value and catch in Figure 8), the cumulative profit that fisheries could make if no subsidies are given is larger than with subsidies regardless of what optimisations are run, i.e. if you wanted to maximise profit the best option would be not to subsidize the fisheries.

Removing subsidies does not make a significant difference on overall ecosystem redundancy in the 7 years of the simulations, as it is very dependent on changes in the lower trophic levels (phytoplankton and zooplankton) which are mainly influenced by changes in the environment [21]. These changes were not included in the optimisation routine, and therefore the secondary production, and redundancy did not change much over the last 7 years of the simulation. Nonetheless, which optimisation function you choose – i.e. maximum profit vs maximum ecological stability does have an impact on the redundancy of the system.

However, removing subsidies does change the structure of the fleet, leading to lower effort for most fleets regardless of which function was optimised (profit or ecological stability). The removal of subsidies increased the biomass of cod, haddock, herring and plaice by 1–3% by the end of the simulation (2010) when optimising for profit and for cod, plaice and sole by between 0.3–1.2% when optimising for ecological stability. These changes are not as noticeable as the difference between optimising for ecological stability and the impact of model uncertainty on these should be investigated in more detail. However, as all scenarios were run with equally uncertain input parameters, these results do show the first indication of the negative impact that subsidies have on the biomass of important fish species, and the profit that can be made from the fisheries. Cumulatively, the profit obtainable from the fishery was lower regardless of whether you want to make more money or want to keep the ecological system stable.

Our simulations indicate that rather than forcing those involved in the fishery into the red, fisheries become more profitable when subsidies are removed, despite a decrease in total revenue due to a loss of financial transfers from the government. Amaliorating for this loss may require some re-distribution of effort among the North Sea fisheries or redistribution to the wider economy. In this situation it would be best to avoid removing subsidies completely at first but to re-direct the funds to ease the transition for those affected by reduced subsidies.

We have shown in this contribution contrasting policies that aim to maximise economic and ecological criteria. Neither are particularly attractive as a policy, the purpose here being to demonstrate in contrasting situations how subsidies influence model predictions of past and future profits. Extending these analyses, we plan to focus attention on more realistic scenarios, which might aim to seek a middle ground between ecological and economic targets. Such analyses ideally requires working with stakeholders and policy makers to define, up front, what might be acceptable scenarios worth investigating and, eventually, implementing. Future work will also include the differences in benefits of subsidies to fishermen from different countries.

## Materials and Methods

An ecosystem model of the North Sea, parameterised and calibrated using time series data of catch and biomass [13], [14] was updated to reflect current information on catches and fleet economics, including the amount of subsidies. The study does not explicitly model fleet behaviour or effort dynamics, but uses fleet size and effort as drivers in the ecological model that forms the basis of the study, and economic data such as cost of fishing and net present value of catches as input, as well as estimating fishing effort in the optimisation scenarios. The model was used to make predictions of possible fishing scenarios to examine the impact of subsidies on the sustainability of the ecosystem and on the socio-economics of the dependent fisheries. Two main changes were made to the model as explained in sections 1.2 and 2 below:

### 1. Model specification

#### 1.1 Ecopath with Ecosim – the model framework

Ecopath with Ecosim (http://www.ecopath.org) is a suite of algorithms used to describe static food webs of ecosystems (Ecopath) and their dynamic interactions (Ecosim) to analyse the impact of exploitation and environmental changes on ecosystem. Ecopath is based on two master equations described in Christensen & Walters [22]: one describing the energy balance and another the production of each functional group in the model. The energy balance of each group is described by:
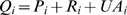
(1)where *Q_i_* is the consumption, *P_i_* the production, *R_i_* the respiration and *UA_i_* the unassimilated food excreted by group *i*. The production of each group is then calculated as:
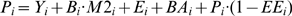
(2)where *P_i_* is the total production of group *i*, *Y_i_* is the total fishery catch rate of *i*, *M2_i_* is the instantaneous predation rate for group *i*, *E_i_* the net migration rate (emigration - immigration), *BA_i_* is the biomass accumulation rate for *i*, and *P_i_·(1−EE_i_)* is the ‘other mortality’ rate for *i* [22].

Ecosim uses the input data from Ecopath as the first timestep in a dynamic expression of biomass through a series of coupled differential equations, where the change in biomass over time is expressed as:
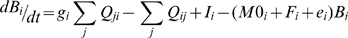
(3)where *dB_i_/dt* is the growth rate during time *t* of group *i* in terms of its biomass *B_i_*; *g_i_* is the net growth efficiency of group *i*; *M0_i_* is the non-predation ‘other’ mortality rate; *F_i_* is the fishing mortality rate; *e_i_* is the emigration and *I_i_* is immigration rate [22]. The *ΣQ_ji_* expresses the total consumption by group *i* and is calculated based on the foraging arena concept, where *B_i_*'s are divided into vulnerable an invulnerable components [23]. *ΣQ_ij_* indicates the predation by all predators of group *i* [24].

Fishing effort is used to calculate the fishing mortality part of total mortality which is used to calculate the biomass of each group in the next time step of the model. The fishing mortality rate *Fi* combined with predation mortality and unexplained mortality *M0i* is used to calculate total mortality in the following formula [25]:
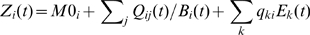
(4)where *M0_i_* is an unexplained natural mortality rate, predation rates *Q_ij_*(*t*) represent total consumption rates of pool *i* by pool *j* predators, and fishing mortality rates *q_ki_E_k_*(*t*) imposed by fishing fleets *k* (including landed catches, by-catch, and dead discards) are represented as varying with time-dependent fishing efforts *E_k_*(*t*) (*k* = 1 …n). Efforts are scaled to 1 in the Ecopath base condition i.e. *E_k_*(*0*) = 1, which allows for the estimation of “catchabilities” *q_ki_* as *q_ki_* = *C_ki_*(*0*)/*B_i_*(*0*) where *C_ki_*(*0*) is an Ecopath base catch of species *i* entered for each fishing effort *k*.

In Ecosim, a formal optimisation routine can be used to evaluate the fishing effort over time that would maximize a particular objective function (or performance measure) as defined by the user [22]. In this analysis we either optimised for net economic value, which optimises the total landed value of the catch minus the total operating costs, or for ecological “stability”, which is measured by assigning a weighting factor to each group based on their longevity, and optimising for the weighted sum [26]. The ecological stability is based on Odum's [15] measure of ecosystem maturity. Ecosim uses the nonlinear Davidson-Fletcher-Powell optimisation procedure to iteratively improve an objective function by changing relative fishing rates, where each fleet defines one parameter (in this case effort) to be varied by the procedure and running the Ecosim model repeatedly while varying these parameters to maximise the objective function [26]. This procedure has been used to describe the trade-offs in fisheries management in systems as varied as the Gulf of Thailand [27] and in the northern Benguela ecosystem [28]. For any further discussion of the parameters and uses of Ecopath with Ecosim see [22], [24], [25].

#### 1.2 Catch profiles of the fisheries

The proportion of the landings and discards of each species taken by each fleet, as reported by STECF (Scientific, Technical and Economic Committee for Fisheries) from 2003 to 2007 [29], was used to update the distribution of landings and discards among the 12 modelled fleets. The STECF does not resolve the catch information to different age groups. Thus, for functional groups split into adult and juvenile components in the model (cod, *Gadus morhua*; whiting, *Merlangius merlangus*; haddock, *Melanogrammus aeglefinus*; saithe, *Polachius virens*; and herring, *Clupea harengus*), the distribution of the catch to landings and discards was maintained as in the original 1991 model [14]. This division is made based on data from discard sampling trips undertaken from 1994–2007. The result of the re-profiling of the distribution of catches is that the model maintains the fishing mortalities of each species in 1991, and hence mass-balance, but is better suited to address the future policy questions addressed here because it reflects the present day fleet structure more accurately.

#### 1.3 Fish prices and fishing costs

Current information on the ex-vessel price (€/tonne) of each species to each fleet and economic performance of each fleet was obtained from the data reported in the 2008 Annual Economic Report [AER, 29] and was used to define the cost and revenue of each modelled fleet and the differences in catch value of each species to each fleet. The data reported in the EAR are mostly taken from the OECD, which uses data provided by the countries themselves [30]. In preparing the data, each modelled fleet was mapped to its corresponding AER fleet (Table S1). The Data Collection Regulations (DCR) provide the basis for this mapping since it is used to define the fleet structures used in both the AER reports and ecosystem model [see 14]. The mapping is however, not a perfect one, with some differences in the fleet descriptions used by the AER, DCR and ecosystem model still remaining. Where AER fleets did not have a direct link to a fleet in the model, the associated catch compositions were examined and used to assign the AER fleet to its corresponding model fleet.

In assigning the prices of each species to the catch of each fleet, we found instances where there was no specific price information for a particular species - fleet combination. Where other price information was available for the species, we assigned the minimum price to that combination; otherwise a nominal value of 1 was assigned (6% of total). We also found a few instances (2% of the total) where price was reported, but there was no catch. These somewhat puzzling cases were confined to shellfish groups and reflect some of the differences in the sources of information arising from AER and STECF [29].

Fixed- and effort-related costs reported for each fleet in the AER include the subsidies paid to the fleets. Costs in the AER report [29] that are classified as fixed or capital costs are defined as fixed cost in Ecopath, while fuel, crew, repair and variable costs in the AER report are all classified as effort-related costs in the model.

#### 1.4 Subsidies

The new fleet structure was used to update subsidies reported for each country in Sumaila et al. [1], where the fixed and variable cost subsidies for each fleet were assumed to be proportional to its share of landed value from the North Sea. For example, if Belgian beam trawlers operating in the North Sea take 1/5^th^ of the value of Belgium's landings, the subsidies to their North Sea beam trawlers are assumed to be 1/5^th^ of Belgium's fishing subsidies. Subsidy types reported in Sumaila et al. [1] are assumed to be focused towards fixed or effort-related (variable) costs as described in Table S2.

This share of subsidies data was used to estimate the proportion of fixed and effort related costs of each fleet that were subsidised, by combining it with the AER cost data to calculate how the gross revenue of each fleet differed when subsidies were included and when they were not (Table S3). Using the information in Table S3, two parameterisations of the ecosystem model were made, one with subsidies included in the costs of fishing, the other without. In the “without subsidies” parameterisation, the costs of fishing are higher, because the calculated proportion of the costs that are subsidised is added to the costs given in the AER data. During simulations, the fixed costs remain constant for the duration of the model simulations (see below). Effort-related costs vary during the simulation depending on the effort of each fleet. In the policy optimisation, subsidies decrease the cost of fishing and therefore when optimising for maximum profit the effort will be increased.

#### 1.5 Understanding fishing profit vs. revenue

Our simulation analysed profit in the North Sea fisheries with and without subsidies. When contrasting profits in the two scenarios, it is important to note that in the scenarios with subsidies, the total revenue generated by a given fishery is augmented by the subsidy, while this does not occur in the non-subsidy case. Since a subsidy represents a government transfer, economically, this is not considered profit generated in a fishery and, as such, subsidies and total costs are subtracted from total revenue to produce an estimate of ‘true’ fishery profit. This measure can then be compared to profit in the non-subsidy scenarios in our simulations.

Thus, in the “with subsidies” scenario, the profit, π, is given by the equation:
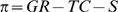
(5)where *GR* is the gross revenue, *TC* is total cost and *S* is subsidies.

The amount of subsidies, S, is calculated as:
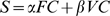
(6)where the parameters *α* and *β* are the subsidised proportions of fixed cost (*FC*) and variable cost (*VC*), respectively.

In the “without subsidies model”, the profit, *π*, is given by the equation:
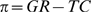
(7)where *GR* and *TC* are gross revenue and total cost as before.

The value of landings is calculated simply as catch*ex-vessel price. In this case, the units for total value are given in millions of €.

### 2. Scenarios

The effects of including or excluding fisheries subsidies were evaluated by performing two types of simulation, namely, Hindcast simulation and Optimisation (2.1 and 2.2).

#### 2.1 Hindcast simulation

The hindcast simulation predicts changes in the relative biomass of each functional group in the model when driven by changes in the fishing effort and mortality, and trends in primary productivity during the period 1991–2003. The simulation has been calibrated to time series data from fish stock assessments and biological surveys by estimating the parameters that influence the strength of the predator-prey interactions. Full details are given in Mackinson et al. [13].

During the simulation, changes in the relative effort of the various fishing fleets were combined to determine the total mortality of the given species. The mortality of a species caused by a particular gear is known as the partial fishing mortality (F), and is calculated as:
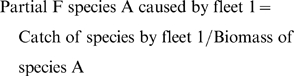
(8)


Because the variable costs of fishing are linked to the amount of fishing effort expended, it is important to have knowledge of how the effort patterns of each fleet changes during the simulation. Trends in effort for each fleet (Figure 1) were obtained from ICES WG assessment reports defined in Table S4.

Hindcast simulations were run with the fixed and variable costs of fishing subsidised and not subsidised. In the non-subsidised version of the model, the costs of fishing where therefore increased so that the real cost of fishing would decrease the profit that is obtained from the fishery. The differences in gross revenue and profit were recorded in millions of €. In addition, subsidies were also removed from the profits calculated by the model post simulation, and these were compared with the scenarios where the subsidies were removed from the value of the fishery as an input variable in the model.

#### 2.2 Optimisation

Two future policy optimisation scenarios were performed (using a Davidson-Fletcher-Powell non-linear routine to improve an objective function by changing relative fishing rates iteratively [27]) to identify:

- The changes in fleet structure of the demersal, beam, pelagic and Nephrops trawls by running 10 optimisations starting from random fishing mortalities (to avoid optimisation being trapped in local minima) for each run to see if the effort distribution is stable;
- What profit can be made from the four different fleets when optimising for a) profit or b) ecological stability;
- The impact that the optimised run would have on the ecosystem, specifically:
  - What changes there would be on the landings and biomass of the principle species (cod, haddock, whiting, Nephrops, plaice, sole, herring, Norway pout); and
  - What changes there would be to fishery stability and ecosystem resilience?

The two policy optimisation scenarios were:

1. maximising economic return, and by contrast;
2. maximising the ecological stability of the ecosystem.

The economic optimisation scenario aims to maximise the total profit (net economic value, i.e. value - fixed and effort related costs), over all fleets even if this means operating some fleets unprofitably to act as controls on less valued species that compete/predate on more valued ones [24]. The ecological stability scenario maximises the longevity-weighted summed biomass over all the ecosystem groups. This index is calculated from the inverse of the production/biomass ratio and the biomass calculated for each group [27].

In addition, future scenarios were run with- and without subsidies. The fitted model was run forward for 7 years from the start of 2004 to the end of 2010 optimising for profit or ecological stability in the last 7 years using 2003 as the base year. Thus the optimisation begins at the end of the period of declines in effort.

The effort of the inshore fisheries were not optimised for, but held constant over the duration of the simulation. The rationale for this is that fisheries policies are aimed at making changes in the main commercial fleets prosecuting fisheries in the central North Sea, whereas, local and regional management decisions are the tools used to affect change in the inshore fisheries.

From the simulations estimates of fishery stability and ecosystem resilience were obtained. The fishery stability is defined by the FiB index [31] calculated for a given year by the formula:
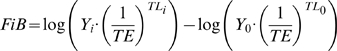
(9)where Y is the catch, TL the mean trophic level in the catch, TE is the transfer efficiency and 0 is the baseline year.

The ecosystem resilience is estimated using the information theory index of redundancy (R), first estimated by Ulanowicz [32] and defined in Ulanowicz [33] as an indicator of the change in degrees of freedom of the system. It is an indicator of the distribution of energy flow among the pathways in the ecosystem, and is calculated as:
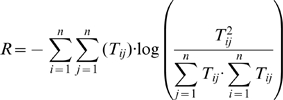
(10)where *T_ij_* is the flow between any two compartments *i* and *j*. These indices and the methodology of getting them from Ecosim are further described in Heymans et al. [21].

## Supporting Information

Table S1 AER and Ecopath model fleet group.
(PDF)

Table S2 Fixed cost and effort-related subsidies by subsidy type.
(PDF)

Table S3 Revenues, costs and profits, with (a) and without (b) subsidies.
(PDF)

Table S4 Sources for effort data used in the hindcast simulations.
(PDF)

Table S5 Catch composition and price of the most important species.
(PDF)
