# Supplementary material for: The Impact of Subsidies on the Ecological Sustainability and Future Profits from North Sea Fisheries
Source: PLoS One. 2011 May 26;6(5):e20239. doi: 10.1371/journal.pone.0020239 (PMC3102685; doi:10.1371/journal.pone.0020239)
Supplement: Table S1 — AER and Ecopath model fleet group. (PDF) [file pone.0020239.s001.pdf]

Table S1: AER and Ecopath model fleet group.

Country	Fleets	Model Fleet group
Belgium	Beam trawl 12m - 24m	Beam trawl
Belgium	Beam trawl 24m - 40m	Beam trawl
Belgium	Demersal trawl and demersal seiner 24m - 40m	Demersal trawl & seine
Belgium	Drift nets and fixed nets 12m - 24m	Drift & fixed nets
Germany	Beam trawl 0m - 12m	Shrimp trawls
Germany	Beam trawl 12m - 24m	Shrimp trawls
Germany	Beam trawl 24m - 40m	Beam trawl
Germany	Demersal trawl and demersal seiner 0m - 12m	Demersal trawl & seine
Germany	Demersal trawl and demersal seiner 12m - 24m	Demersal trawl & seine
Germany	Demersal trawl and demersal seiner 24m - 40m	Demersal trawl & seine
Germany	Drift nets and fixed nets 12m - 24m	Drift & fixed nets
Germany	Non Active Vessels 0m - 12m	Non Active Vessels
Germany	Non Active Vessels 12m - 24m	Non Active Vessels
Germany	Non Active Vessels 24m - 40m	Non Active Vessels
Germany	Non Active Vessels over 40m	Non Active Vessels
Germany	Passive gears 0m - 12m	Drift & fixed nets
France	Beam trawl 0m - 12m	Beam trawl
France	Beam trawl 12m - 24m	Beam trawl
France	Beam trawl 24m - 40m	Beam trawl
France	Combining mobile and passive gears 0m - 12m	Dredges
France	Combining mobile and passive gears 12m - 24m	Dredges
France	Combining mobile and passive gears 24m - 40m	Other methods
France	Demersal trawl and demersal seiner 0m - 12m	Demersal trawl & seine
France	Demersal trawl and demersal seiner 12m - 24m	Nephrops trawls
France	Demersal trawl and demersal seiner 24m - 40m	Demersal trawl & seine
France	Demersal trawl and demersal seiner over 40m	Demersal trawl & seine
France	Dredges 0m - 12m	Dredges
France	Dredges 12m - 24m	Dredges
France	Dredges 24m - 40m	Dredges
France	Drift nets and fixed nets 0m - 12m	Drift & fixed nets
France	Drift nets and fixed nets 12m - 24m	Drift & fixed nets
France	Drift nets and fixed nets 24m - 40m	Drift & fixed nets
France	Drift nets and fixed nets over 40m	Drift & fixed nets
France	Gears using hooks 0m - 12m	Gears using hooks
France	Gears using hooks 12m - 24m	Gears using hooks
France	Gears using hooks 24m - 40m	Gears using hooks
France	Other mobile gears 0m - 12m	Other methods
France	Other mobile gears 12m - 24m	Other methods
France	Other passive gears 0m - 12m	Dredges
France	Other passive gears 12m - 24m	Other methods
France	Pelagic trawls and seiners 0m - 12m	Pelagic trawl & seine
France	Pelagic trawls and seiners 12m - 24m	Pelagic trawl & seine
France	Pelagic trawls and seiners 24m - 40m	Pelagic trawl & seine
France	Pelagic trawls and seiners over 40m	Pelagic trawl & seine
France	Polyvalent mobile gears 0m - 12m	Dredges
France	Polyvalent mobile gears 12m - 24m	Dredges
France	Polyvalent mobile gears 24m - 40m	Pelagic trawl & seine
France	Polyvalent passive gears 0m - 12m	Drift & fixed nets
France	Polyvalent passive gears 12m - 24m	Other methods
France	Pots and traps 0m - 12m	Pots
France	Pots and traps 12m - 24m	Pots
GBR	Beam trawl 0m - 12m	Beam trawl

GBR	Beam trawl 12m - 24m	Beam trawl
GBR	Beam trawl 24m - 40m	Beam trawl
GBR	Beam trawl over 40m	Beam trawl
GBR	Combining mobile and passive gears 0m - 12m	Other methods
GBR	Combining mobile and passive gears 12m - 24m	Other methods
GBR	Demersal trawl and demersal seiner 0m - 12m	Nephrops trawls
GBR	Demersal trawl and demersal seiner 12m - 24m	Nephrops trawls
GBR	Demersal trawl and demersal seiner 24m - 40m	Demersal trawl & seine
GBR	Demersal trawl and demersal seiner over 40m	Demersal trawl & seine
GBR	Dredges 0m - 12m	Dredges
GBR	Dredges 12m - 24m	Dredges
GBR	Dredges 24m - 40m	Dredges
GBR	Dredges over 40m	Dredges
GBR	Drift nets and fixed nets 0m - 12m	Drift & fixed nets
GBR	Drift nets and fixed nets 12m - 24m	Drift & fixed nets
GBR	Drift nets and fixed nets 24m - 40m	Drift & fixed nets
GBR	Drift nets and fixed nets over 40m	Drift & fixed nets
GBR	Gears using hooks 0m - 12m	Gears using hooks
GBR	Gears using hooks 12m - 24m	Gears using hooks
GBR	Gears using hooks 24m - 40m	Gears using hooks
GBR	Non Active Vessels 0m - 12m	Non Active Vessels
GBR	Non Active Vessels 12m - 24m	Non Active Vessels
GBR	Non Active Vessels 24m - 40m	Non Active Vessels
GBR	Non Active Vessels over 40m	Non Active Vessels
GBR	Pelagic trawls and seiners 0m - 12m	Pelagic trawl & seine
GBR	Pelagic trawls and seiners 12m - 24m	Pelagic trawl & seine
GBR	Pelagic trawls and seiners 24m - 40m	Pelagic trawl & seine
GBR	Pelagic trawls and seiners over 40m	Pelagic trawl & seine
GBR	Polyvalent mobile gears 0m - 12m	Other methods
GBR	Polyvalent mobile gears 12m - 24m	Other methods
GBR	Polyvalent mobile gears 24m - 40m	Other methods
GBR	Polyvalent mobile gears over 40m	Other methods
GBR	Polyvalent passive gears 0m - 12m	Other methods
GBR	Polyvalent passive gears 24m - 40m	Other methods
GBR	Pots and traps 0m - 12m	Pots
GBR	Pots and traps 12m - 24m	Pots
GBR	Pots and traps 24m - 40m	Pots
Netherlands	Beam trawl 0m - 12m	Beam trawl
Netherlands	Beam trawl 12m - 24m	Beam trawl
Netherlands	Beam trawl 24m - 40m	Beam trawl
Netherlands	Beam trawl over 40m	Beam trawl
Netherlands	Demersal trawl and demersal seiner 0m - 12m	Demersal trawl & seine
Netherlands	Demersal trawl and demersal seiner 12m - 24m	Demersal trawl & seine
Netherlands	Demersal trawl and demersal seiner 24m - 40m	Demersal trawl & seine
Netherlands	Dredges 0m - 12m	Dredges
Netherlands	Dredges 24m - 40m	Dredges
Netherlands	Dredges over 40m	Dredges
Netherlands	Non Active Vessels 0m - 12m	Non Active Vessels
Netherlands	Non Active Vessels 12m - 24m	Non Active Vessels
Netherlands	Non Active Vessels 24m - 40m	Non Active Vessels
Netherlands	Non Active Vessels over 40m	Non Active Vessels
Netherlands	Other passive gears 0m - 12m	Other methods
Netherlands	Other passive gears 12m - 24m	Other methods
Netherlands	Other passive gears 24m - 40m	Other methods

Netherlands	Pelagic trawls and seiners 0m - 12m	Pelagic trawl & seine
Netherlands	Pelagic trawls and seiners 12m - 24m	Pelagic trawl & seine
Netherlands	Pelagic trawls and seiners over 40m	Pelagic trawl & seine
Netherlands	Polyvalent passive gears 0m - 12m	Drift & fixed nets
Netherlands	Polyvalent passive gears 12m - 24m	Other methods
Netherlands	Polyvalent passive gears 24m - 40m	Other methods
Sweden	Demersal trawl and demersal seiner 0m - 12m	Nephrops trawls
Sweden	Demersal trawl and demersal seiner 12m - 24m	Nephrops trawls
Sweden	Demersal trawl and demersal seiner 24m - 40m	Demersal trawl & seine
Sweden	Drift nets and fixed nets 12m - 24m	Drift & fixed nets
Sweden	Gears using hooks 12m - 24m	Gears using hooks
Sweden	Non Active Vessels 0m - 12m	Non Active Vessels
Sweden	Non Active Vessels 12m - 24m	Non Active Vessels
Sweden	Passive gears 0m - 12m	Other methods
Sweden	Pelagic trawls and seiners 12m - 24m	Pelagic trawl & seine
Sweden	Pelagic trawls and seiners 24m - 40m	Pelagic trawl & seine
Sweden	Pelagic trawls and seiners over 40m	Pelagic trawl & seine
Denmark	Beam trawl 12m - 24m	Shrimp trawls
Denmark	Beam trawl 24m - 40m	Beam trawl
Denmark	Demersal trawl and demersal seiner 0m - 12m	Demersal trawl & seine
Denmark	Demersal trawl and demersal seiner 12m - 24m	Nephrops trawls
Denmark	Pelagic trawls and seiners 12m - 24m	Pelagic trawl & seine
Denmark	Pelagic trawls and seiners 24m - 40m	Pelagic trawl & seine
Denmark	Pelagic trawls and seiners over 40m	Pelagic trawl & seine
Denmark	Dredges 0m - 12m	Dredges
Denmark	Dredges 12m - 24m	Dredges
Denmark	Polyvalent passive gears 0m - 12m	Other methods
Denmark	Polyvalent passive gears 12m - 24m	Other methods
Denmark	Combining mobile and passive gears 0m - 12m	Other methods
Denmark	Combining mobile and passive gears 12m - 24m	Other methods
