# Supplementary material for: The Impact of Subsidies on the Ecological Sustainability and Future Profits from North Sea Fisheries
Source: PLoS One. 2011 May 26;6(5):e20239. doi: 10.1371/journal.pone.0020239 (PMC3102685; doi:10.1371/journal.pone.0020239)
Supplement: Table S2 — Fixed cost and effort-related subsidies by subsidy type. (PDF) [file pone.0020239.s002.pdf]

Table S2: Fixed cost and effort-related subsidies by subsidy type.

Subsidy type	Predicted effect of subsidy	Effort-related or fixed cost subsidy
Boat construction & modernization	Capacity enhancing	Fixed cost
Development projects	Capacity enhancing	Fixed cost
Port construction	Capacity enhancing	Fixed cost
Marketing & storage support	Capacity enhancing	Effort-related
Tax exemptions	Capacity enhancing	Effort-related
Fuel subsidy	Capacity enhancing	Effort-related
Fisher assistance programs	Ambiguous	Effort-related
Vessel buyback programs	Ambiguous	Fixed cost
Rural development programs	Ambiguous	Fixed cost
