# Supplementary material for: The Impact of Subsidies on the Ecological Sustainability and Future Profits from North Sea Fisheries
Source: PLoS One. 2011 May 26;6(5):e20239. doi: 10.1371/journal.pone.0020239 (PMC3102685; doi:10.1371/journal.pone.0020239)
Supplement: Table S3 — Revenues, costs and profits, with (a) and without (b) subsidies. (PDF) [file pone.0020239.s003.pdf]

Table S3: Revenues, costs and profits, with (a) and without (b) subsidies.

		(a) Subsidies included (%)				Subsidies				(b) Without subsidies (subsidies added back on to costs, %)			
Fleet	Average of Total Income (million Euro)	Total cost	Fixed cost	Variable cost	Revenue	Average of Subsidized portion of fixed costs	Average of subsidized portion of variable costs	Fixed cost subsidy	Variable cost subsidy	Total cost	Fixed cost	Variable cost	Revenue
Demersal trawl & seine	33.9	32.4	8.5	23.8	1.5	0.30	0.04	2.56	0.91	35.8	11.1	24.8	-2.0
Beam trawl	31.1	31.4	10.3	21.1	-0.4	0.15	0.03	1.57	0.73	33.7	11.9	21.9	-2.7
Sandeel trawl	0.0	0.0	0.0	0.0	0.0	0.00	0.00	0.00	0.00	0.0	0.0	0.0	0.0
Pelagic trawl & seine	40.9	38.4	13.3	25.1	2.5	0.26	0.03	3.50	0.65	42.6	16.8	25.8	-1.7
Drift & fixed nets	17.2	16.7	3.3	13.3	0.6	0.52	0.02	1.74	0.29	18.7	5.1	13.6	-1.4
Nephrops trawls	101.1	98.3	24.6	73.7	2.8	0.32	0.03	7.89	1.93	108.1	32.5	75.6	-7.0
Gears using hooks	8.5	7.5	1.4	6.1	1.0	0.14	0.00	0.19	0.00	7.7	1.6	6.1	0.8
Shrimp trawls	20.2	20.4	7.4	13.0	-0.2	0.14	0.11	1.01	1.49	22.9	8.4	14.5	-2.7
Dredges	13.6	12.6	3.0	9.6	1.0	0.10	0.03	0.31	0.28	13.2	3.3	9.9	0.4
Shellfish picking	0.0	0.0	0.0	0.0	0.0	0.00	0.00	0.00	0.00	0.0	0.0	0.0	0.0
Pots	25.9	22.2	5.5	16.7	3.7	0.14	0.00	0.79	0.01	23.0	6.3	16.7	2.9
Other methods	2.9	2.4	0.7	1.7	0.5	0.59	0.06	0.38	0.11	2.9	1.0	1.8	0.0
