# Supplementary material for: The Impact of Subsidies on the Ecological Sustainability and Future Profits from North Sea Fisheries
Source: PLoS One. 2011 May 26;6(5):e20239. doi: 10.1371/journal.pone.0020239 (PMC3102685; doi:10.1371/journal.pone.0020239)
Supplement: Table S4 — Sources for effort data used in the hindcast simulations. (PDF) [file pone.0020239.s004.pdf]

Table S4: Sources for effort data used in the hindcast simulations.

Fleet	Period available	Source and notes
Demersal trawl & seine	1978-2008	(WGNNSK08, 2008), including fleets SCOSEI_IV, SCOLTR_IV, ENGTRL_IV, ENGSEI_IV, FRATRB_IV, FRATRO_IV, NORTRL_IV, GER_OTB_IV
Beam trawl	1979-2007	(WGNNSK08, 2008), including fleets NL_BT_EFF,UK_BT_EFF
Pelagic trawl & seine	1987-2006	(WGNNSK08, 2008) fleet NOR_DEN_NPOUT_EFF
Nephrops trawl	1981-2004	(WGNSSK06, 2006), summed over Nephrops functional units
Shrimp trawl	1984-2003	(WGPAN, 2005), Pandulus – total international effort in ICES div IV.
