# Supplementary material for: The Impact of Subsidies on the Ecological Sustainability and Future Profits from North Sea Fisheries
Source: PLoS One. 2011 May 26;6(5):e20239. doi: 10.1371/journal.pone.0020239 (PMC3102685; doi:10.1371/journal.pone.0020239)
Supplement: Table S5 — Catch composition and price of the most important species. (PDF) [file pone.0020239.s005.pdf]

Table S5: Catch composition and price of the most important species.

	Proportion of catch				Price			
**Group Name**	**Demersal**	**Beam**	**Pelagic**	**Nephrops**	**Demersal**	**Beam**	**Pelagic**	**Nephrops**
Starry ray + others	0%	2%	0%	0%	1.81	1.81	1.81	0
Cod (adult)	5%	12%	0%	8%	1.97	1.49	1.94	1.84
Whiting (adult)	9%	5%	1%	37%	1.48	0.9	0.9	0.9
Haddock (adult)	9%	1%	0%	17%	1.38	1.17	1.17	1.17
Saithe (adult)	16%	0%	0%	4%	0.82	0.82	0.82	0.82
Blue whiting	2%	0%	3%	0%	0.15	0	0.15	0
Norway pout	1%	0%	13%	0%	1	0	1	0
Other gadoids (large)	3%	0%	0%	2%	2.3	2.14	4.07	2.14
Monkfish	1%	1%	0%	5%	4.07	3.23	3.23	4.07
Gurnards	0%	2%	0%	0%	1.25	1.25	1.25	1.25
Herring (adult)	40%	0%	33%	0%	0.2	0.2	0.28	0.25
Sprat	0%	1%	10%	0%	0.14	0.14	0.21	0.16
Mackerel	5%	0%	27%	0%	0.71	0.71	1.06	0.71
Horse mackerel	0%	0%	9%	0%	0.37	0.37	0.37	0.37
Plaice	1%	30%	0%	3%	1.9	1.92	1.79	1.82
Flounder	1%	20%	0%	0%	0.41	0.74	0.41	0
Sole	0%	19%	0%	0%	10.06	9.75	11.17	10.22
Lemon sole	0%	2%	0%	2%	4.01	4.01	6.34	4.01
Witch	1%	0%	0%	6%	3.8	3.8	3.8	3.8
Nephrops	0%	0%	0%	10%	6.63	3.1	9.17	7.2
	**96%**	**95%**	**97%**	**95%**				
